# Using Nodal Ratios to Predict Risk of Regional Recurrences in Patients Treated with Breast Conservation Therapy with 4 or More Positive Lymph Nodes

**DOI:** 10.5402/2011/874814

**Published:** 2011-06-30

**Authors:** William Castrucci, Donald Lannin, Bruce G. Haffty, Susan A. Higgins, Meena S. Moran

**Affiliations:** ^1^Department of Therapeutic Radiology, Yale University School of Medicine, 333 Cedar Street, P.O. Box 208040, New Haven, CT 06520-8040, USA; ^2^Backus Breast Center and Department of Radiation Therapy, USA; ^3^Department of Surgical Oncology, Yale University School of Medicine, 333 Cedar Street, P.O. Box 208040, New Haven, CT 06520-8040, USA; ^4^Department of Radiation Oncology, UMDNJ-Robert Wood Johnson School of Medicine, and the Cancer Institute of New Jersey, New Brunswick, NJ 08903, USA

## Abstract

*Purpose*. The value of nodal ratios (NRs) as a prognostic variable in breast cancer is continually being demonstrated. The purpose of this study was to use NR in patients with ≥4+ nodes to assess a correlation of NR with regional (lymph node) recurrence. *Methods*. Inclusion criteria was ≥8 nodes dissected with ≥4+ nodes after breast conservation therapy. Of 1060 patients treated from 1975 to 2003 who had a minimum of 8 nodes dissected, 273 were node+; 56 patients had ≥4+ involved nodes and were the focus of this study. Nodal ratios were calculated for each patient and grouped into 3 categories: high (≥70%), intermediate (40%–69%) and low (<40%). Each nodal ratio was correlated with patterns of local, regional, and distant failures and OS. *Results*. Outcomes for the entire cohort were BRFS-83%, NRFS-93%, DMFS-61%, and OS 63% at 10 yrs. The OS, DMFS, and NRFS correlated with N2 (4–9 nodes+) versus N3 (≥10+) status but did not correlate with BRFS, as expected. When evaluating NR, 18 pts had high NR (>70%). Only 3 patients experienced nodal recurrences, all within previously radiated supraclavicular fields. All 3 in-field regional failures occurred in the N3 group of patients with NR >70%. All were treated with a single AP field prescribed to a dose of 46 Gy at a standard depth of 3 cm. *Conclusions*. In this group of N2/N3 patients treated with BCT, we were able to identify patients at high risk for regional failures as those with high NR of >70% and ≥10+ nodes. While these findings need to be reproduced in larger datasets, this group of patients with NR of >70% in 4 or more positive axillary lymph nodes may benefit from meticulous targeting of regional nodes, dose escalation, and/or more intensive systemic therapies.

## 1. Introduction

Surgical pathologic evaluation of axilla is a standard part of the management of breast-conserving therapy for breast cancer; this has both prognostic and therapeutic implications. While the presence of axillary lymph metastasis is the single most critical predictor of survival in breast cancer, the absolute number of lymph nodes involved appears to be equally as important. To reflect the importance of this fact, the 6th edition of the AJCC/TNM Cancer Staging Manual incorporated major changes in the pathologic nodal staging for breast cancer to include the number of lymph nodes involved (1–3+, 4–9+, ≥10+) so that an increasing number of positive nodes results in higher N-stage and overall stage [1]. 

Potentially confounding the value of “number of lymph nodes involved” is the variability of total number of lymph nodes excised or examined during axillary staging. As a result, there has been increasing interest in the use of the nodal ratio as a possible prognostic factor. The nodal ratio (NR) is defined as the absolute number of positive nodes identified divided by the total number of nodes removed and has been used to potentially eliminate the confounding of “involved” versus “examined” nodes. The nodal ratio has significant prognostic value for locoregional recurrence and survival, with some authors suggesting that it may be even more important than the absolute number of positive nodes [2]. 

Although rare, supraclavicular recurrences are difficult to salvage and are often associated with a poor prognosis [3]. The nodal ratio has not previously, to our knowledge, been used to predict the risk of in-field regional recurrence after BCT. The purpose of our study was to determine if, in the high-risk patient population who have undergone adequate axillary dissection, nodal ratios can help identify a subgroup of patients that are at highest risk for nodal in-field recurrences so that radiation treatment modifications can potentially be made to improve regional relapse rates in this high-risk population.

## 2. Materials and Methods

After obtaining IRB approval, the medical records of patients with breast cancer undergoing breast-conserving surgery and whole breast radiation therapy at Yale University School of Medicine treated from 1975 to 2003 were reviewed. The present study cohort consisted of a group of high-risk, early-stage, invasive breast cancer patients who had a minimum of 8 lymph nodes excised with breast-conserving surgery followed by adjuvant radiation therapy (*n* = 1060). Of this group, 273 patients were lymph-node positive, and of these, 56 patients (20.5%) had ≥4 positive nodes. These 56 patients comprised the study cohort for the current investigation.

After conservative surgery, all patients were treated with radiation therapy using standard isocentric techniques using 6 MV photons with appropriate wedges delivering a median dose of 48 Gy to the entire breast. An electron cone down was utilized in all patients to bring the total median dose to the tumor bed to 64 Gy. Regional nodal radiation was delivered at the discretion of the treating physician, in accordance to the standard practices of our institution during the time period in which the patients were treated. The institutional policy has generally been not include the axilla after ALND unless there is an inadequate number of lymph nodes excised, or significant residual tumor burden in the axilla (as estimated by pathology or surgical/operative report), due to the increased risk of lymphedema. Typically, the supraclavicular fossa was treated to a median dose of 46 Gy for ≥1 involved axillary node. The regional nodal basins irradiated were evaluated as a function of nodal ratio. 

The technique for regional nodal radiation has been previously described [4] and is only briefly discussed here. External beam radiation was administered to the supraclavicular fossa using a separate anterior beam half-blocked at the central axis. The field was angled 10–15 degrees off of the spinal cord and extended from midline medially (at the skin surface) to the medial border of the humeral head laterally. If the residual burden of disease in the axillary region was thought to be high, the lateral border was extended laterally to beyond the humeral head to encompass the axilla. Because these patients were treated in an era in which CT-simulation was not routinely performed, the majority of the patients underwent traditional, fluoroscopic simulation, and the supraclavicular field dose was specified to a standard depth of 3 cm in all patients.

Nodal ratios (NRs) were calculated for each patient as follows: absolute number of lymph nodes involved divided by the number of lymph nodes excised, multiplied by 100 (expressed as a percentage). Patients were grouped into three categories: low (NR < 40%), intermediate (NR < 70% and ≥40%), and high (NR ≥ 70%). 

Breast relapse-free survival was defined by time to local failure in the treated breast. Nodal relapse-free survival was time to regional failure (axilla, supraclavicular fossa, infraclavicular fossa, or internal mammary nodes). Disease free survival was defined by time to disease failure outside of the local/regional area. All events were calculated from the time of initial diagnosis to the time of the event. The outcomes were calculated using standard life-table methods.

The nodal ratio for each patient and relevant covariables were assembled in a database and analyzed using PRODAS, Version 9.1 (SAS Institute, Cary, NC). All tests of statistical significance were two sided. *P* values less than  .05 were considered statistically significant.

## 3. Results

As of September 2006, the median followup time was 7 years. Of the 56 patients in our cohort, 29% (*n* = 16) were in the low, 39% (*n* = 22) in the intermediate, and 32% (*n* = 18) in the high NR groups, respectively. The clinical characteristics of the entire cohort, as a function of nodal ratio distribution, are given in Table 1. 

Table 2 describes the radiation field delivery as a function of nodal ratio. Of note, 4 patients of the 56 in our cohort had only tangential breast radiation fields without any regional nodal radiation. These patients were classified in the low nodal ratio group (*n* = 3) and the intermediate nodal ratio group (*n* = 1). Thus, the remainder of the 52 patients in the cohort received supraclavicular radiation (± axillary radiation); specifically, all patients in the high nodal ratio group received supraclavicular radiation to a median dose of 46 Gy. As shown in Table 3, none of the patients in the low NR group had more than 9 positive nodes identified on axillary dissection, whereas 67% of patients in the High NR group had 10 or more positive nodes identified. 

For the entire cohort, the overall survival was 62%, disease-free survival was 61%, breast relapse-free survival was 83%, and nodal relapse-free survival was 93% at 10 years. The nodal ratio was predictive of nodal relapse free survival (77% in the HNR group versus 100% in the LNR and INR groups (*P* < .05). The OS, DMFS, and NRFS correlated with N2 (4–9 nodes+) versus N3 (≥10+) status but did not correlate with BRFS, as expected. While there was a trend toward poorer overall survival and disease-free survival in the HNR group, the differences were not statistically significant.

Three of the 56 patients had regional nodal relapses; all 3 nodal recurrences were in the supraclavicular fossa in patients with high nodal ratios (Table 4). Therefore, the 7% nodal relapse rate was due solely to supraclavicular relapses, with no axillary or internal mammary relapses. All 3 regional recurrences had been treated with a single AP field prescribed to a dose of 46 Gy at 3 cm, as described above. In this group of patients with high nodal ratios, the regional relapse rate in the treated supraclavicular fossa at 5 years was 35%.

## 4. Discussion

The absolute number of positive lymph nodes dissected from the axilla is currently recognized as the most significant predictor of survival in breast cancer [5–8]. It is intuitive that the fewer the number of lymph nodes removed in dissection of the axilla, the less reliable this predictor will be, with significant potential for understaging and under treating. Exactly what constitutes an “adequate” axillary dissection, however, in terms of providing sufficient prognostic and therapeutic benefits, remains a topic of considerable debate. In contemporary series of patients with early breast cancer, when 7–10 lymph nodes are identified in the axillary specimen, fewer than 3% of patients will experience axillary failure [3, 9, 10]. Most studies assessing the extent of dissection required to accurately assess nodal status suggest that >10 nodes should be examined [11]. To a certain extent, the variation in the total numbers of “axillary nodes removed” between series of patients reflects not only differences in surgical technique, but also in the pathologists' examination of the specimens. 

The concept of the nodal ratio was developed in order to reduce the confounding of the number of involved lymph nodes by the extent of surgical clearance and thoroughness of pathologic examination. By combining the “involved” and “examined” nodes into a single value, the prognostic implications of axillary dissection may be more reliable and may potentially lead to better selection of patients requiring adjuvant regional nodal therapy. In addition, for comparison of different series of patients, the nodal ratio can serve as a uniform standard value. Nodal ratio has been particularly useful when looking at patients with 1–3 positive nodes and/or inadequate dissections, as these have historically been groups for whom the decision to use adjuvant radiation therapy is considered controversial.

Outcomes are worse with higher numbers of positive nodes and with higher nodal ratios, with a number of studies suggesting that the latter is the stronger of the two prognostic factors. The nodal ratio has been shown to be predictive for breast cancer outcomes in a variety of clinical scenarios (e.g., early-stage and locally advanced, mastectomy and BCT, ± chemotherapy, and/or radiation) [8, 11–17]. These studies have found nodal ratios to be predictive of a variety of outcome parameters including locoregional relapse, disease-free survival (DFS), progression-free survival (PFS), cause-specific survival (CSS), overall survival (OS), and metastasis-free survival. Furthermore, nodal ratios have also been used to rectify interinstitutional differences in outcomes that exist due to variations in axillary dissection practice [18]. These findings are reviewed in a recent comprehensive publication of the prognostic value of nodal ratios [2]. 

Among the studies that have used nodal ratio as a prognostic indicator, various thresholds have been used for definition of a “high” ratio. Our definition of high nodal ratio was similar to the publications by Tai et al., the International Nodal Ratios Working Group, and others, that used ratios of >50%–75% for their high nodal ratio group [2, 11]. In the above-mentioned review of nodal ratios that included >20 clinical studies, the percentage cutoffs for nodal ratios varied depending upon the outcomes being investigated and the level of statistical significance being sought [2]. Hence, there has yet to be established a clear benefit to the use of any one method of grouping patients by nodal ratio over any other.

Our study suggests that high nodal ratios of >70% are predictive of supraclavicular relapse in women with early-stage breast cancer who have had at least 8 nodes excised on ALND. It is important to note that while the vast majority of these patients received supraclavicular radiation, the fields were determined clinically in the era in which these patients were treated, with the dose prescribed routinely at a depth of 3 cm, without CT guidance, as was the convention at our institution. More recent investigations in the CT treatment planning era suggest that the depth of the supraclavicular nodes varies significantly, ranging from 2.4–9.5 cm (median = 4.3 cm) [19]. Given this variability in depth and the substantial risk of supraclavicular recurrence in the high nodal ratios risk group, meticulous attention should be paid by the treating radiation oncologist to contouring the location of the supraclavicular lymph nodes with careful selection of the radiation beam energy, with consideration given to a supraclavicular boost in high-risk patients to ensure optimal delivery of dose to these lymph nodes at potential risk for recurrence.

## 5. Conclusions

While supraclavicular fossa recurrences are rare after breast conservation therapy, our data suggest that in patients with 4 or more positive axillary nodes treated with traditional tangential/supraclavicular techniques, high nodal ratios (>70%) predict for in-field regional recurrences. While these findings need to be reproduced in larger datasets, more meticulous treatment planning of regional nodes, radiation dose escalation, and/or more intensive systemic therapies in high nodal ratio patients should be considered.

##  Conflict of Interests

The authors declared that there is no conflict of interests to disclose.

## Figures and Tables

**Table 1:** Patient characteristics as a function of nodal ratio.

Clinical parameter	LNR *n* (%)	INR *n* (%)	HNR *n* (%)	Total *n* (100%)
Patient age				
<50 years	3 (15)	11 (52)	7 (33)	21
≥50 years	13 (38)	11 (31)	11 (31)	35

Tumor size				
T1	6 (21)	11 (39)	11 (39)	28
T2	10 (36)	11 (39)	7 (25)	28

Pathologic subtype				
Invasive ductal	16 (31)	20 (38)	16 (31)	52
Invasive lobular	0 (0)	1 (50)	1 (50)	2
Other	0 (0)	1 (50)	1(50)	2

Estrogen status				
Positive	9 (35)	11 (42)	6 (23)	26
Negative	4 (19)	8 (38)	9 (43)	21

Progesterone status				
Positive	10 (39)	11 (42)	5 (19)	26
Negative	3 (17)	6 (33)	9 (50)	18

Chemotherapy				
Yes	10 (63)	20 (91)	16 (89)	46
No	6 (37)	2 (9)	2 (11)	10

Abbreviations/definitions: LNR = low nodal ratio <40%; INR = Intermediate Nodal Ratio, 40%–70%; HNR = High Nodal Ratio >70%.

**Table 2:** Radiation treatment field as a function of nodal ratio.

Ratio	Tangents alone *n* (%)	Tangents + SC *n* (%)	Total *n* (%)
LNR	3 (5)	13 (23)	16 (28)
INR	1 (2)	21 (38)	22 (40)
HNR	0 (0)	18 (32)	18 (32)

Total	4 (100)	52 (100)	56 (100)

Abbreviations/definitions: LNR = Low nodal ratio <40%; INR = Intermediate Nodal Ratio, 40%–70%; HNR = High Nodal Ratio >70%. Tangents alone: no nodal radiation intentionally delivered. Tangents + SC*: Tangents with supraclavicular field ± IM/axilla, as clinically indicated. Percentages given as a fraction of the total cohort (*n* = 56).

**Table 3:** Number of involved nodes on axillary dissections as a function of nodal ratio.

	Number of involved lymph nodes		
	4–9 + nodal group *n*	≥10 + nodal group *n*	Total *n*
LNR	16	0	16
INR	14	8	22
HNR	6	12	18
Total	36	20	56

Abbreviations/definitions: LNR = low nodal ratio <40%; INR = intermediate nodal ratio, 40%–70%; HNR = high nodal ratio >70%. *n* = number of patients in each nodal group. Percentages given as a fraction of the total cohort (*n* = 56).

**Table 4:** Nodal recurrence as a function of nodal ratio.

Ratio	Axilla *n* (%)	SC *n* (%)	IM *n* (%)	Total *n* (%)
LNR	0 (0)	0 (0)	0 (0)	0 (0)
LNR	0 (0)	0 (0)	0 (0)	0 (0)
HNR	0 (0)	3 (100)	0 (0)	3 (100)

Total	0 (0)	3 (100)	0 (0)	3 (100)

Abbreviations/definitions: LNR = low nodal ratio <40%; INR = intermediate Nodal Ratio, 40%–70%; HNR = high nodal ratio >70%. SC = supraclavicular recurrence; IM = internal mammary recurrence. All patients with nodal relapse received supraclavicular radiation. Percentages given as a fraction of total nodal relapses (*n* = 3).
